# What Kind of Doctor Do You Want to Be? Geriatric Medicine Podcast as a Career Planning Resource

**DOI:** 10.1155/2017/6183148

**Published:** 2017-06-19

**Authors:** Anna Byszewski, Kathryn Bezzina, Meriem Latrous

**Affiliations:** ^1^Department of Medicine, Division of Geriatrics, University of Ottawa, Ottawa, ON, Canada; ^2^Faculty of Medicine, University of Ottawa, Ottawa, ON, Canada

## Abstract

**Introduction:**

For optimal direction in career paths and postgraduate training, students can benefit from information to guide them through options. Using geriatric medicine as a template, the goal was to develop a multimedia podcast resource that can give a clearer picture of what a specialty entails.

**Methods:**

The project included a survey of existing resources and needs assessment of medical students at the University of Ottawa, Canada. This survey assessed students' knowledge of geriatrics and interest in the field and explored what they foresee as being important to be informed on when considering application to programs. Based on this, interview questions and content were developed for a podcast which was then evaluated.

**Results:**

Interviews were conducted with physicians and residents nationwide. Relevant resources and links were added to the podcast. Evaluation demonstrated improved student understanding and interest in geriatric medicine as a career. Point-by-point format for a template on how to develop similar podcasts was developed to assist other specialties looking to develop similar information.

**Conclusions:**

As no such framework currently exists, results of this project can serve as a template for other postgraduate programs in developing a multimedia resource for informing prospective trainees.

## 1. Introduction

A recent Canadian Royal College of Physicians and Surgeons report highlighted areas of medicine that are overserviced but also emphasized those that are underserviced, including geriatric medicine [1].

In 2015 United Nations reported that the population of those over 80 is expected to quadruple by 2050 reaching 400 million. To meet the complex care of the growing population of older persons, increasing numbers of physicians need to develop expertise in geriatric medicine [2]. Despite this increasing demand, knowledge of and interest in geriatrics as a career choice remain low. In 2008 the US reported that 44% of spots for training in geriatrics were left unfilled [3]. In Canada we do not have such published data.

A recent systematic review [4] was conducted to evaluate literature on some of the factors involved in this lack of uptake of geriatric medicine as a career choice. The data base identified 2164 records but once duplicates were removed and full texts excluded, for reasons including studies not pertaining to medical students and low quality scores, 20 studies met criteria for the qualitative synthesis. Based on this review, a major factor for career choice included a preference for looking after younger patients with acute medical conditions that could be easily cured. Some of the other factors dissuading choice of this specialty involved the complexity of the geriatric patient, dealing with chronic diseases and a short life expectancy. The lack of status and positive role models, as well as perceived or relative lower financial remuneration, was also cited as factors contributing to geriatrics being unattractive. But once students have had an opportunity to manage these conditions, their attitude may change [5]. A sense of reward and further incentive to develop expertise often ensues.

The systematic review also indicated that having previous exposure to the elderly had a positive effect on considering this career choice, especially if it was positive, and if students saw themselves as care-takers in providing quality of life and comfort. Some of the studies indicated that students would be discouraged if they saw futility in solving problems and seeing this as a personal failure. The complexity of care and ambiguity was viewed as overwhelming to some students. However others found the focus on the entire patient intellectually stimulating and saw opportunities for research. Recommendations to increase knowledge and interest included ensuring students have exposure to geriatric patients and geriatric care, as studies show the positive effect of such experiences [5–8]. The systematic review [4] also indicated that studies confirm that students are influenced by medical school characteristics, such as values and culture of a particular institution, the curriculum content, and faculty structure. The authors emphasize the importance of faculty demonstrating rewarding aspects of their practice, guidance on complex patient management, dealing with ethical issues and ambiguity, and focusing on a patient-orientated approach, instead of disease-orientated approach.

In Canada, recent reports indicate that 1 in 6 medical specialists cannot find work [9]. This is partly explained by the fact that the medical school enrollment in Canada has almost doubled over the last 20 years. This, coupled with the “baby boomer” generation delaying retirement, has led to some specialties being overprescribed in Canada's publicly funded health care system. Residents may find themselves without positions when they are ready to enter practice and generally report a lack of adequate career counselling and information about jobs [1].

On the other hand, students indicate that few of them seek career counselling guidance, mostly obtaining advice from family, other students, and mentors. They may thus be unaware of opportunities and challenges in the marketplace in the future. When considering future job opportunities, lifestyle and intellectual stimulation remain the top motivators for choice of specialty as well as previous exposure and patient contact [10, 11]. Ensuring that there are opportunities for exposure to different patient populations and practice settings may allow future physicians to make informed choices.

The Association of American Medical Colleges (AAMC) has led an initiative called the Careers in Medicine program which provides some guidance where students can read the profiles of more than 120 specialties and subspecialties; however at this time it is only in text form [12].

Given these gaps in information and career guidance, as well as low uptake of geriatric medicine [1], the goal of this project was to develop a template for a multimedia podcast resource that would be readily available and that would inform about career choices in a given specialty, using geriatric medicine as a template.

## 2. Materials and Methods

**Figure 1:**
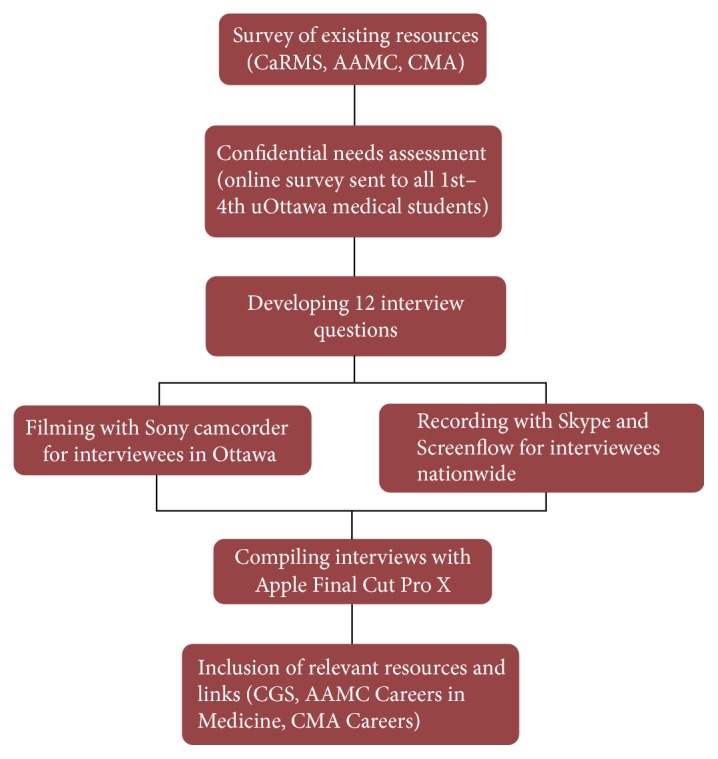
Materials and methods.

Various national and international medical websites were explored as well as organizations such as the Association of Faculties of Medicine of Canada (AFMC), Canadian Medical Association (CMA), Canadian Federation of Medical Students (CFMS), and Association of American Medical Colleges (AAMC) for career guidance information (Figure 1). Most are still text based and provide a general overview of specialty descriptions. A survey was conducted with the University of Ottawa medical students on what they saw being important for prospective trainees to be informed on. In Canada, in order to meet the needs of the growing population of older persons, there are two routes of training: the Geriatric Medicine Residency program (two years following the core internal medicine three-year program) and Care of the Elderly program (one year following the two-year family medicine program). Based on the survey, a 12-question podcast interview questionnaire was developed. Key physicians leaders and trainees in both Geriatric Medicine and the Care of the Elderly program were contacted across the country to participate.

The podcast was developed using a Sony Camcorder for recording interviews locally in Ottawa, while Skype and ScreenFlow were used to interview and record nationwide.

The video podcast is 7.5 minutes long and consists of the following:

- Input from several Geriatric Medicine and Care of the Elderly residents and physician leaders across Canada.
- Themes addressed:
  - the definition of geriatrics;
  - what is attractive about the specialty;
  - the daily tasks and responsibilities of a geriatrician;
  - geriatrics in the future;
  - job market opportunities;
  - training paths, research, and teaching opportunities;
  - important personality traits needed for success in the specialty;
  - suggestions on how to prepare for an application to a geriatrics residency program and how to learn more about the specialty.

The interviews were compiled using Apple Final Cut ProX and vital links were added, including AFMC, CMA, and the Canadian Geriatrics Society websites. The podcast is available at https://www.youtube.com/watch?v=wu87dZOr1xk&feature=youtu.be. It is currently housed on the Canadian Geriatric Society and the University of Ottawa ThinkOttawaMedicine websites.

The video podcast was presented to the second-year medical students at a nonmandatory class during the start of a two-week introductory course to geriatrics. A brief 13 question pre- and postquestionnaire was administered electronically to establish the impact of the podcast on students' understanding of a geriatric medicine career and their interest in pursuing a career in geriatric medicine.

This project was approved by Ottawa Health Science Network Research Ethics Board (OHSNREB).

## 3. Results

The response rate to the evaluation of the podcast at the geriatrics introductory lecture was 60.4% (93/154). As lectures are nonmandatory at the University of Ottawa, thus not all of the 154 second-year medical students would have been present to view the podcast, affecting the response rate.

Table 1 demonstrates the demographics of the students responding; 61.5% were female and 61.5% were less than 25 years old. Regarding prior geriatric experience, 62.6% had no prior exposure to geriatrics.

The results of the impact of the podcast on students' knowledge and considering a career in geriatrics are presented in Table 2. Before the podcast 42.9% of students rated their knowledge of what a career in geriatrics may entail, as very little or none. This decreased to 9.9% after podcast, thus indicating improved understanding. Overall, after viewing the podcast, the knowledge of what a career in geriatrics is like, improved significantly (*p* < 0.0001). The* p* value of 0.0001 was derived with Fisher's exact test.

As well, after viewing the podcast more students indicated that they might consider a geriatrics career, although this did not reach significance (*p* = 0.1729), using Fisher's exact test. Before the podcast 48.4% were “perhaps considering” or “thinking about” a geriatrics residency. This shifted to 59.4% after viewing the video, with 2 students (2.2%) saying they would “definitely” consider a career in geriatrics.

The pre- and postresponses were also analyzed using multinomial logistic regression using GEE (generalized estimating equation) models adjusting for previous experience in geriatric medicine. There were statistically significant differences between pre- and postresponses. The odds of responding positively after the podcast was 5.5 higher than the odds in prepodcast for the question “How much do you know about a career in geriatric medicine?” As well, the odds of responding positively after the podcast was 1.6 higher than prepodcast for the question “How likely would you consider a career in geriatric medicine?”

**Figure 2:**
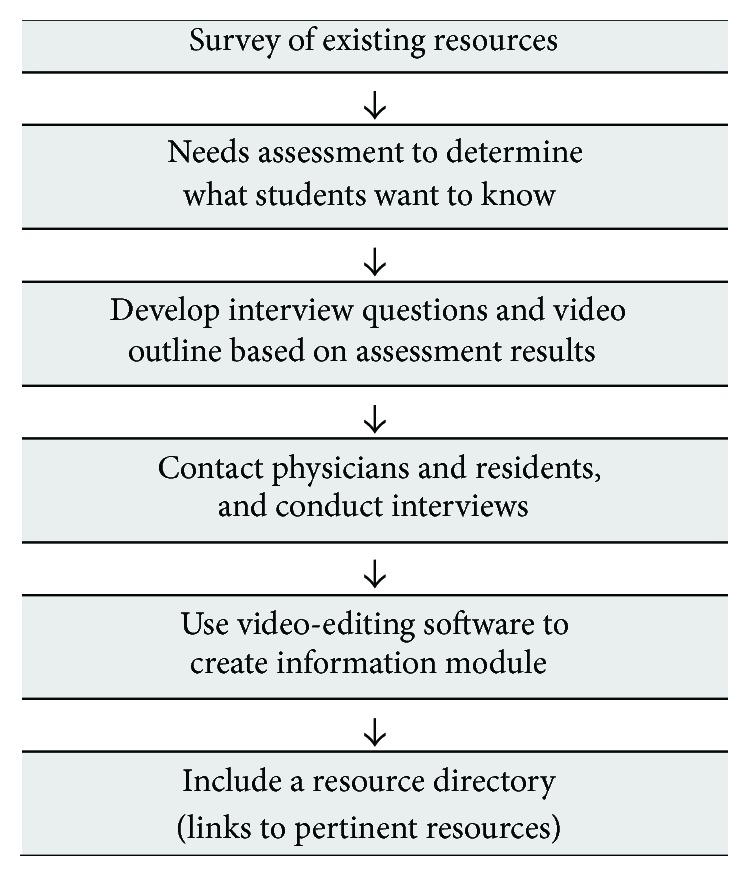
Template for creating career planning podcasts.

Other suggestions from students, included in Box 1, indicate some recommendations on how to enrich the delivery of the information. Students found the podcast as a creative method to present a specialty and suggested creating podcasts for other medical fields. Comments also included suggestions for including a clip on “A day in the life of… (geriatrician etc)…” and involving patient testimonials. These suggestions will be incorporated in our future podcast iterations. A template demonstrating steps useful for developing career planning podcasts is included in Figure 2. This includes a needs assessment of themes and strategies to include in the podcast as well as links to resources students can explore.

## 4. Discussion

Table 1 shows demographics of the students, with most having no experience in geriatrics. On viewing the podcast, medical students were more aware of what a career in geriatric medicine was, with a significant “average amount” of knowledge, increasing from 14.3% before to 39.6% after viewing the podcast (Table 2). There was also a trend for more students considering geriatric medicine as a career choice, following the viewing of the podcast, with 2 students definitely considering this career. Even with a short intervention such as a podcast, we were able to demonstrate impact on student awareness of the field of geriatrics and spark some interest in this area. To generate more commitment to pursuing a career in geriatrics, real experience in a clinical setting would be necessary. Not surprisingly, those students who had some previous exposure to geriatrics had a 5.5 higher odds of responding positively in the postpodcast than the odds in prepodcast questionnaire, to the question “How much do you know about a career in geriatric medicine?” As well for these students, the odds of responding positively after the podcast was 1.6 higher than prepodcast to the question “How likely would you consider a career in geriatric medicine?” This result suggests the impact of having prior exposure to geriatrics in medical training and potential for greater impact when presented with more information on career opportunities in geriatric medicine from engaged faculty and other trainees.

Geriatric medicine is a fairly new specialty across the world, with the first recognition of this area of internal medicine being recognized by Dr. Marjorie Warren in the UK. In the 1950s she implemented a model of rehabilitation for hospitalized elderly patients and encouraged their mobilization. Being a relative young discipline, the first geriatric programs in the United States were established in the late 1970s, with the first separate department of geriatrics founded in 1982 at Mount Sinai School of Medicine. In North America, most medical schools have a geriatric curriculum and most medical schools world-wide are increasing geriatric content in their curricula. However, as a career choice, there are still gaps in knowledge, awareness and uptake as a profession. With the current demographics and increasing complexity of patients, with a multitude of comorbidities including cognitive decline, a comprehensive geriatric assessment is gaining momentum, now often acknowledged as the “tool” of geriatrics. As role modeling is often recognized as being essential for career direction, multimedia resources can provide sources of information to those seeking information on a career in geriatrics, particularly in programs where there may not be an active geriatric specialty training unit. Some of the barriers recognized include the prevailing competition for subspecialty curricula content, ageist attitudes, and lack of positive experiences [4]. To address these obstacles and to meet the needs of the aging population, the specialty needs to attract future leaders in this discipline.

Multimedia learning enhancements to garner student engagement can be utilized to support “authentic learning,” by captivating interest and involving the observer in the subject matter [13]. This online podcast provides an overview of the different paths for gaining expertise and outlines opportunities to develop different interests, such as medical education, community practice, and academic paths, as well as emphasizing complexities of the field, the rewards of interdisciplinary care, and satisfaction knowing that one can make a difference to our seniors.

In order to assist with career planning, medical schools are developing more focused interventions to aid students such as University of Michigan Medical School career development program (CDP) [14]. This is a 4-phase career planning program, which includes self-assessment exercises, guidance from counsellors and shadow experiences with faculty and senior students. A UK career support strategy has been developed with a 4-stage approach to career planning and a checklist for medicine on track (MOT) for health professionals [15]. The template that we have developed and present here can serve as a guide for other areas of medicine that can adapt and develop similar resources to enhance their programs to aid trainees and program directors.

## 5. Limitations

The objective of this project was to develop and evaluate the impact of a novel multimedia career planning intervention, with a template that could be adapted in other fields outside of geriatric medicine. We did not examine specifically what was the impact of different prior experiences in geriatrics, such as electives, rotations, courses, or personal life experiences with seniors, as it was not the primary objective of this study. Our response rate was 60.4%, limited by students responding in a lecture setting, which are nonmandatory at our institution. This project was conducted at one university setting with a brief intervention. Similar projects will need to be conducted in diverse settings to validate our findings and enhance such program delivery.

## 6. Conclusions

This current podcast can be utilized to raise awareness of geriatric medicine as a potential career choice, which may also encourage other less competitive fields, especially when undersubscribed, to consider for stimulating trainee interest. In addition, this template for podcasts as presented (Figure 2) can serve as a novel multimedia approach demonstrating career options for medical students considering a variety of residency program options.

## Figures and Tables

**Box 1:**
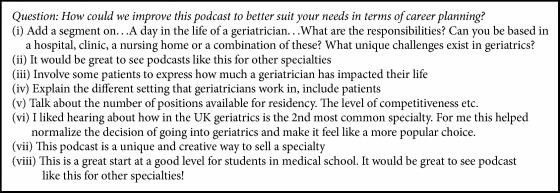
Student comments.

**Table 1:** Demographics.

Demographics (*n* = 93)	
Gender	
Female	61.5%
Male	38.5%
Age	
<25	61.5%
25–29	33%
30–34	5.5%
35+	0.0%
Previous geriatric experience	
Geriatric experience	37.4%
No geriatric experience	62.6%

**Table 2:** Podcast evaluation (*N* = 93/154).

How much do you know about a career in geriatric medicine? (*p* < 0.0001)					
*Pretest*			*Posttest*		
Nothing	3.3%	3	Still nothing	0.0%	0
Very little	39.6%	36	Very little	9.9%	9
Some	41.8%	38	Some	47.3%	43
Average amount	14.3%	13	Average amount	39.6%	36
A lot	1.1%	1	A lot	3.3%	3

How likely would you consider a career in geriatric medicine? (*p* = 0.1729)					
*Pretest*			*Posttest*		
Definitely not	17.6%	16	Definitely not	11.0%	10
Not sure	34.1%	31	Not sure	27.5%	25
Perhaps	26.4%	24	Perhaps	33.0%	30
Thinking about it	22.0%	20	Thinking about it	26.4%	24
Definitely yes	0.0%	0	Definitely yes	2.2%	2
